# Design of hybrid narrow-band plasmonic absorber based on chalcogenide phase change material in the infrared spectrum

**DOI:** 10.1038/s41598-021-01479-w

**Published:** 2021-11-09

**Authors:** Israel Alves Oliveira, Igor Leonardo Gomes de Souza, Vitaly Felix Rodriguez-Esquerre

**Affiliations:** 1 Graduate School of Electrical Engineering, Federal University of Bahia, Salvador, 40210-630 Brazil; 2 Institute of Science, Technology and Innovation at the Federal University of Bahia (ICTI-UFBA), Camaçari, 42802-721 Brazil

**Keywords:** Nanoscience and technology, Nanoscale devices, Nanophotonics and plasmonics

## Abstract

Structures absorbing electromagnetic waves in the infrared spectral region are important optical components in key areas such as biosensors, infrared images, thermal emitters, and special attention is required for reconfigurable devices. We propose a three-dimensional metal-dielectric plasmonic absorber with a layer of PCM’s (Phase Change Materials). The phase shift effects of PCMs are numerically analyzed, and it is possible to obtain a shifting control of the resonant absorption peaks between the amorphous and crystalline states using the Lorentz–Lorenz relation. By using this empirical relation, we analyzed the peak absorption shift at intermediate phases between the amorphous and the crystalline. The geometric parameters of the structure with the PCM layer in the semi-crystalline state were adjusted to exhibit strong absorption for normal incidence. The effects of the oblique incidence on the absorption for the TM and TE polarization modes were also analyzed. Our results demonstrate that PCMs have great potential for reconfigurable nanophotonic devices.

## Introduction

Electromagnetic absorber structures based on metamaterials and plasmonics that are able to achieve almost perfect electromagnetic absorption, are very important devices because of their wide applications, such as detection^1,2^, solar energy^3–5^, selective thermal emitters of wavelength^3^ and, radiation cooling^4^. To date, there are several reports of absorbers operating over different bands of the electromagnetic spectrum. Most of them are based on a Metal–Dielectric–Metal configuration, having specific absorption bandwidths, restricting their high performance applications^5–7^. Recent studies demonstrated an extraordinary absorption response from the Bi-based MPA in ultra-broadband, polarization insensitive, and almost omnidirectional NIR (near infrared) perfect absorber by using Bi nanodiscs in an metal–isolator–metal configuration^8^. The GST chalcogenide phase change materials, (this includes GeTe), can be quickly^9^ and repeatedly^10^ switched between amorphous and crystalline states by appropriate thermal, electric, or optical stimuli^11–13^. Until now, GST chalcogenides have been regarded as strong candidates for realizing reconfigurable and nonvolatile all-optical devices^14^, consequently, several applications have been proposed, such as all-optical switching^15^, optical filters^16^, and reconfigurable metasurfaces^17,18^. Plasmonic absorbers based on PCMs including GeTe were analyzed in Refs.^19–21^, this resonant structures composed by metals and PCM's as insulators with sub-wavelength dimensions have been used as mirrors to suppress transmission by increasing the reflection and inducing destructive interference from reflected light at resonant wavelengths absorbed on the structure^22^. These proposed works explore the great contrast of the material's optical constants between its amorphous and crystalline phases in the regions of the electromagnetic spectrum from the visible (VIs) to the infrared (IR).

VO_2_ based PCM devices have been used earlier and they allow resonance tuning when combined with metallic structures. In this way, thermal reconfigurable absorbers have been also investigated. However, the metallic phase imposes a lower temperature limit of 68 °C being considered a volatile material^23,24^. Non volatile PCM could be obtained when chalcogenides are used. Nowadays, one of the most used materials is the Ge_2_Sb_2_Te_5_ (GST 225). Plasmonic and hybrid metamaterial reconfigurable absorbers have been proposed using the GST225^25–27^. Several chalcogenides have been used in the infrared region (Ge_3_Sb_2_Te_6_, Ge_2_Sb_2_Te_4_, Ge_2_SbTe_4_, Ag_4_em_3_Sb_67_Te_26_ e GeTe) They are deposited over polar substrates (SiC, Al_2_O_3_ e SiO_2_) exhibiting high refractive index contrast between the amorphous phase, where they are lossless and transparent, while the crystalline phase exhibits high attenuation. Consequently, the absorption occurs in the substrate for the amorphous phase while for the crystalline phase it happens in the PCM layer^28^. Also, PCMs based in In_3_SbTe_2_ (IST) which can switch from dielectric to metallic states are also designed to operate in the infrared region^29^.

GeTe has a crystallization temperature higher than the GST, exhibiting also different transition times which are needed for NVM^30,31^ applications an additional practical applications^32,33^. Although the above mentioned advantages of the GeTe, there are still few absorbers based on this material. Our results demonstrate the feasibility of using GeTe for the design of reconfigurable devices.

The main characteristic of PCMs is that they are not volatile at room temperature and can change gradually between phases (amorphous, crystalline or intermediate) in a periodic way with billions cycles per second (nanosecond or faster time response) by applying an external stimulus, which can be optical, temperature variation or electrical voltage^9,34^. The Induced crystallization of GeTe can be done using laser pulses that give the material enough heat for the phase transition. In this type of stimulus, only a small section of the GeTe crystallizes, i.e. the area where the laser hit the material, limiting the number of possible applications. The transition from amorphous (a-GeTe) to crystalline (c-GeTe) phase in GeTe using conduction heating is completely reversible and happens after a heating of approximately 200 °C, in this phase change there is a difference of approximately six orders in the magnitude of its resistivity. This characteristic can be used for data storage devices^35^. The transition between phases can also be done using short and high intensity voltage pulses (joule heating), these pulses use DC sources, an advantage of this external stimulus is the fast switching between states approximately 10 ns from the a-GeTe state to c-GeTe, and 5 ns from the c-GeTe state to a-GeTe^36^.

Although some works on GST-based absorbers have been published in the literature, the proposed one in this work demonstrates an alternative and ideal solution for designing reconfigurable absorbers based on GeTe PCM’s with dielectric substrate. We propose a novel absorber for applications in optical communications with absorption above 95%, with values of 98% in the second and third optical communications window covering the O + E + S + C bands.

The proposed absorber is reconfigurable in the range spectrum of 1100–1900 nm. Using the effective medium theory, we explored the crystallization fractions of GeTe to tune the resonance peak of the absorber. We also evaluated the behavior of absorbers for oblique angles of incidence (up to 45°). Here we propose some possibilities to tune the resonant peak of this absorber: (1) with the crystallization fraction; (2) with the geometric parameters. In addition, we also evaluated the effects of the absorption with the variation of the geometrical parameters and the physical absorption mechanisms through the analysis of the spatial distribution of the electromagnetic fields and the current density inside the absorber.

## Structure’s design and methods

**Figure 1:**
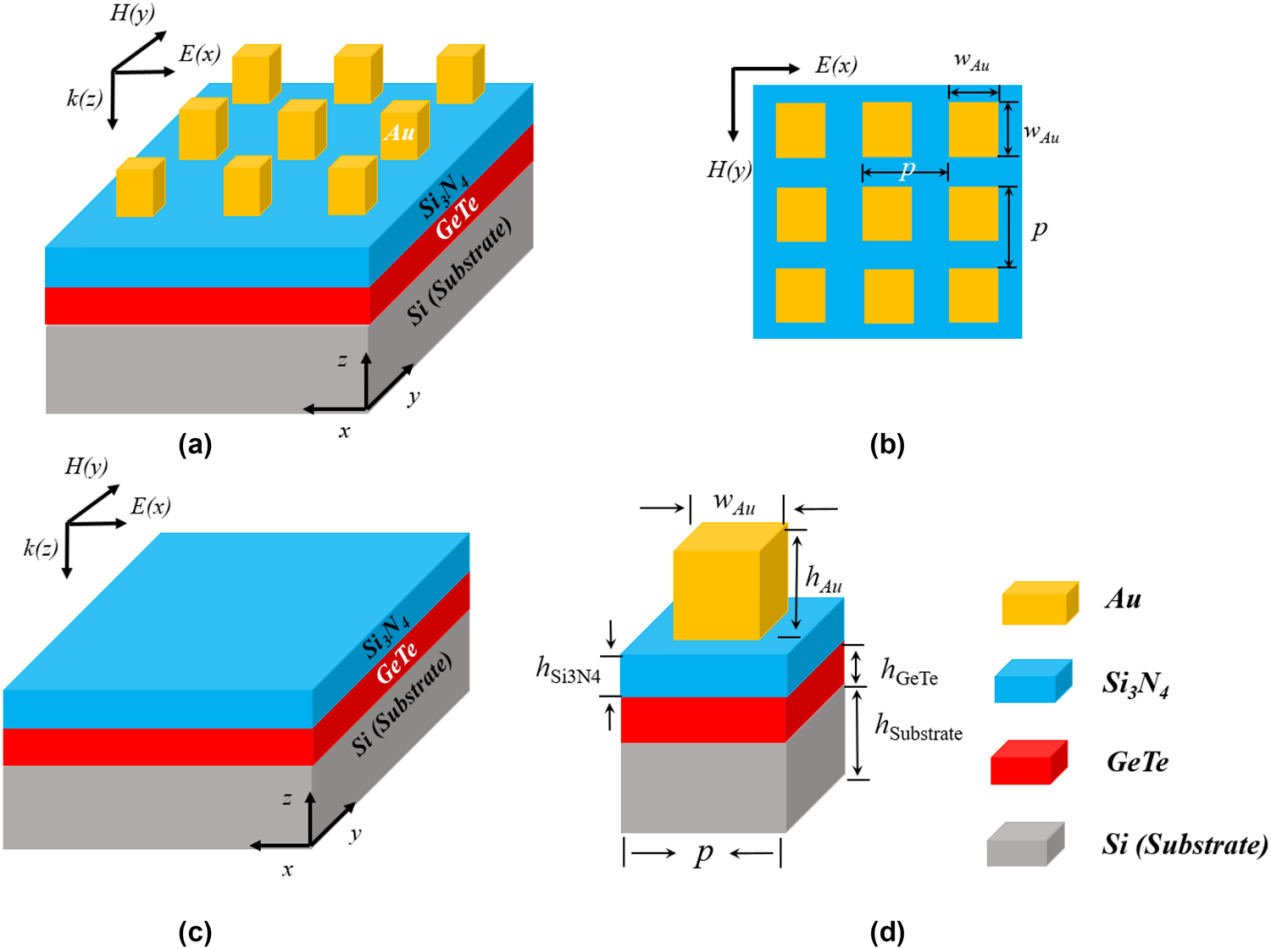
(**a**) Schematic representation of the hybrid absorber; (**b**) top view of the absorber the hybrid plasmonic absorber; (**c**) representation of the planar control absorber without Au nanoantennas; (**d**) unit cell of the hybrid plasmonic absorber.

**Figure 2:**
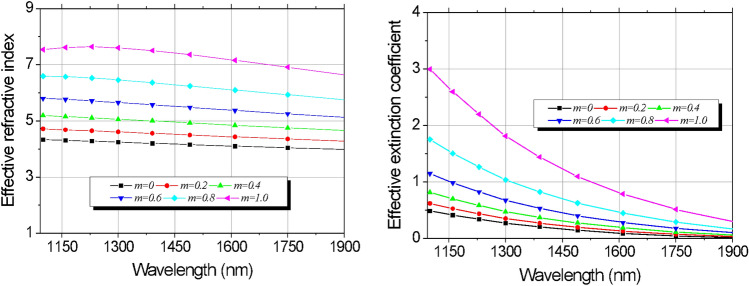
(**a**) Refractive index and; (**b**) extinction coefficient of the GeTe using Eq. (1).

In this study, a hybrid, tunable and reconfigurable perfect absorber in the infrared spectral range considering a plasmonic metamaterial using a chalcogenide phase change material (GeTe) is proposed and theoretically investigated. Our nanoplasmonic structure consists of a periodic array placed directly on the surface of uniform silicon (Si). The building blocks of our design consist of square gold (Au) nanoantennas above a dielectric Si_3_N_4_ spacer layer and a GeTe layer (Fig. 1a,b). In comparison with the planar absorber (Fig. 1c), the hybrid device with a phase change material can dramatically enhance the absorbance due to the induced electric and magnetic modes at the resonance. For the analysis of the absorber behavior and simulations, we will use the unit cell (Fig. 1d) which represents the geometric parameters of the structure. The Au square nanocubes have two geometric parameters, h_Au_ and w_Au_ with 150 nm and 160 nm, respectively. The insulator spacer (Si_3_N_4_) and GeTe mirror thicknesses are 125 nm and 130 nm, respectively. The whole structure resides on a silicon substrate with 145 mm thickness. The lattice constant in both x- and y-directions is p = 300 nm. The relative permittivity of gold can be obtained through the Drude-Lorentz dispersion^37^. The refractive indexes of the insulators (Si_3_N_4_ and Si) in the analyzed spectrum region are 2.0 and 3.5, respectively. The optical constants of GeTe are explained in Ref.^12^ and they are shown in Fig. 2a,b. The numerical method to calculate the absorption coefficient of the structure is the finite element method (FEM)^38^, using the commercial software COMSOL^39^. The periodic boundary conditions are used for the lateral surfaces and PMLs (perfectly absorbing layers) are applied along the *z* direction to eliminate undesired boundary reflections. Since the substrate film is thicker than the penetration depth of the incident light, it will block the propagation, consequently, the transmittance (*T*) of the structure is almost zero, resulting in the absorption equation *A* = 1* − R*, with R being the reflection). In particular, the fast phase transition in PCMs occurs gradually, and the switching speed is considerably high^40^ rather than suddenly (time responses of about nanoseconds and picoseconds), increasing the possibility of adjustments. During the phase transition, the PCM film can be assumed to be in the intermediate phase composed by different regions of amorphous and crystalline atoms and this phase change kinetics can be accomplished by heating^41^.

Based on the optical constants of the PCM in both phases and using the theory of the effective medium^42^, it is possible to obtain the permittivity at intermediate levels of crystallization for this material. This relation is called Lorentz–Lorentz, as follows:

1 $$\frac{{\upvarepsilon_{eff} (\uplambda ) - 1}}{{\upvarepsilon_{eff} (\uplambda ) + 2}} = m \cdot \frac{{\upvarepsilon_{{{\text{crystalline}}}} (\uplambda ) - 1}}{{\upvarepsilon_{{{\text{crystalline}}}} (\uplambda ) + 2}} + (1 - m) \cdot \frac{{\upvarepsilon_{{{\text{amorphous}}}} (\uplambda ) - 1}}{{\upvarepsilon_{{{\text{amorphous}}}} (\uplambda ) + 2}}$$

where λ is the operation wavelength, $\varepsilon_{eff}$ is the permittivity of the GeTe at the crystallization level *m*, and, $\upvarepsilon_{{{\text{crystalline}}}}$ and, $\upvarepsilon_{{{\text{amorphous}}}}$ are permittivity of GeTe in fully crystallized and fully amorphous states, respectively. We use the relations:

2 $$\varepsilon (\lambda ) = n^{2} (\lambda ) - k^{2} (\lambda )$$

3 $$\tilde{\varepsilon }(\lambda ) = - 2nk$$

In order to calculate the real and imaginary parts of the GeTe permittivity, next, we could determine the $\upvarepsilon_{eff} (\uplambda )$ for each fraction of crystallization by using Eq. (1). The effective refractive index and extinction coefficient are shown in Fig. 2a,b, respectively, they depend on the wavelength and on the levels of crystallization *m*.

## Results and discussions

**Figure 3:**
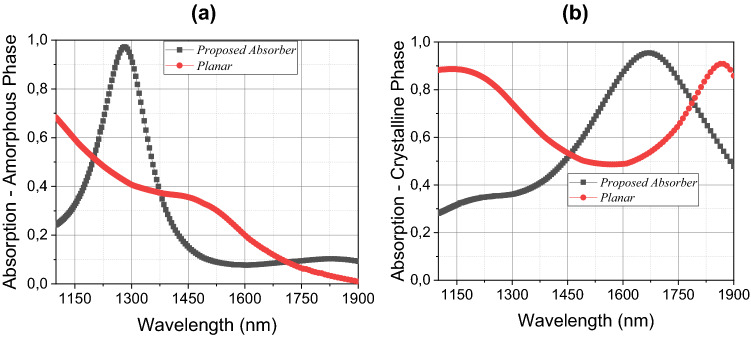
Absorption spectrum of the proposed absorber compared to the planar metadevice on phases: (**a**) amorphous and; (**b**) crystalline.

With the structure properly designed, we compared the results with a continuous planar control device, where the golden layers are absent (see Fig. 1a,c). These results can be seen in Fig. 3a,b. The proposed structure exhibits high absorption in both phases (amorphous and crystalline) at different wavelength peaks (1279 nm and 1664 nm, respectively), while the planar structure (without golden squares) presents a sharp drop in absorption when the length of wave shifts to the right and in the crystalline state, the absorption decreases in the intermediate range of the analyzed spectrum, and again reaches 90% absorption at the wavelength near 1900 nm. Structures based on planar absorbers are ideal for Fabry Perot resonators^19,43,44^, where the conditions of maintaining resonance within the cavity are present. For the proposed structure in this article, cavity resonances can be obtained by using periodic array geometry.

**Figure 4:**
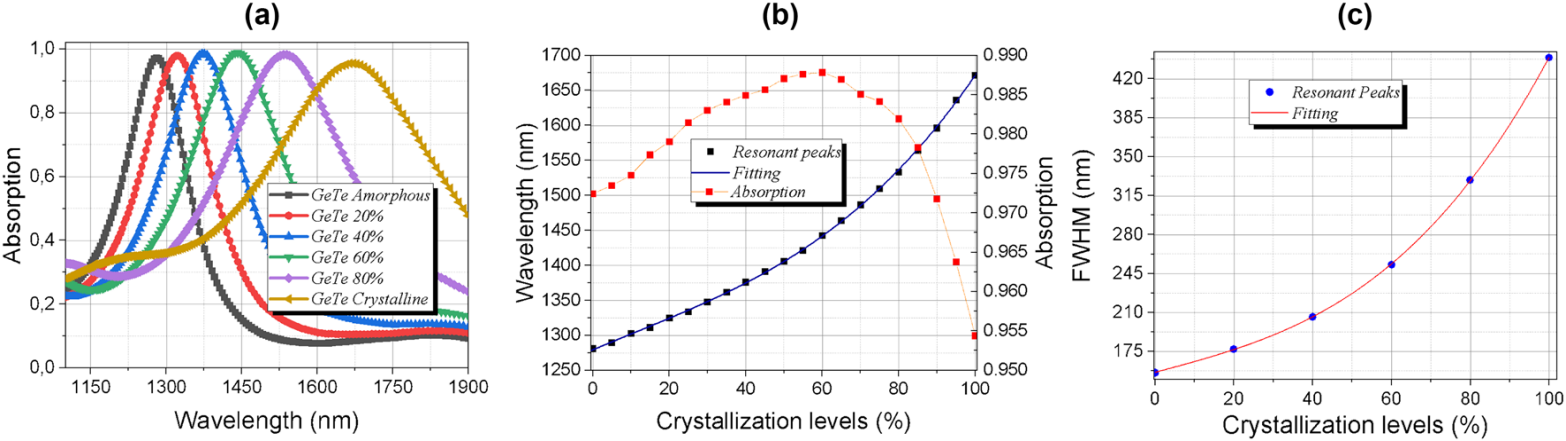
(**a**) The evolution of the absorption spectrum for different crystallization levels (**b**) by a fit polynomial of degree 3. The percentages shown in the legend correspond to the GeTe crystallization levels (**c**) Variation of the Full Width Half Maximum (FWHM) as a function of the crystallization of GeTe, by a fit also polynomial of degree 3.

The Fig. 4a shows the evolution of the absorption spectrum as PCM crystallization level increases for normal incidence. The infrared region of the spectrum is sensitive to phase changes due to the strong electronic polarizability of GeTe in the crystalline state, producing a high contrast in its refractive index^45^. We also numerically analyzed the behavior of the resonant peak position and value with the crystallization fraction (Fig. 4b). The red curve shows the absorption coefficient for each peak, it is possible to analyze that the structure has an absorption above 95.5% for all resonant peaks. The blue curve shows that the first peak is shifted to the right when the crystallization fraction of GeTe increases. Using high precision statistical methods, we predict a function to obtain the resonance peak as a function of the crystallization fraction of GeTe, as follow:

4 $$\uplambda (m_{\% } ) = 1279.15 + 2.32\;m - 7.87 \cdot 10^{ - 2} \;m^{{2}} + 2.38 \cdot 10^{ - 4} \;m^{3}$$

where, *m* is the crystallization fraction of GeTe. This equation can be used to modulate any resonance peak as a function of *m* in that interval of 1100–1900 nm.

The results shown in Fig. 4a revealed an increasing in the FWHM, along the range of crystallization of GeTe. The peak gets wider because the extinction coefficient (k) of gold varies almost linearly as the wavelength increases, resulting in absorption in a larger region of wavelengths, consequently, the FWHM increases. Analogously to obtaining the behavior of this parameter, this evolution can be expressed by the equation as follows:

5 $$FWHM\;(m_{\% } ) = 156.15 + 9.5 \cdot 10^{ - 1} \;m - 3.97 \cdot 10^{ - 8} \;m^{2} + 1.89 \cdot 10^{ - 4} \;m^{3}$$

where, *m*_%_ is the crystallization fraction of GeTe. This equation can be used to analyze the FWHM for any fraction of crystallization of GeTe. Figure 4c shows the behavior of the FWHM.

**Figure 5:**
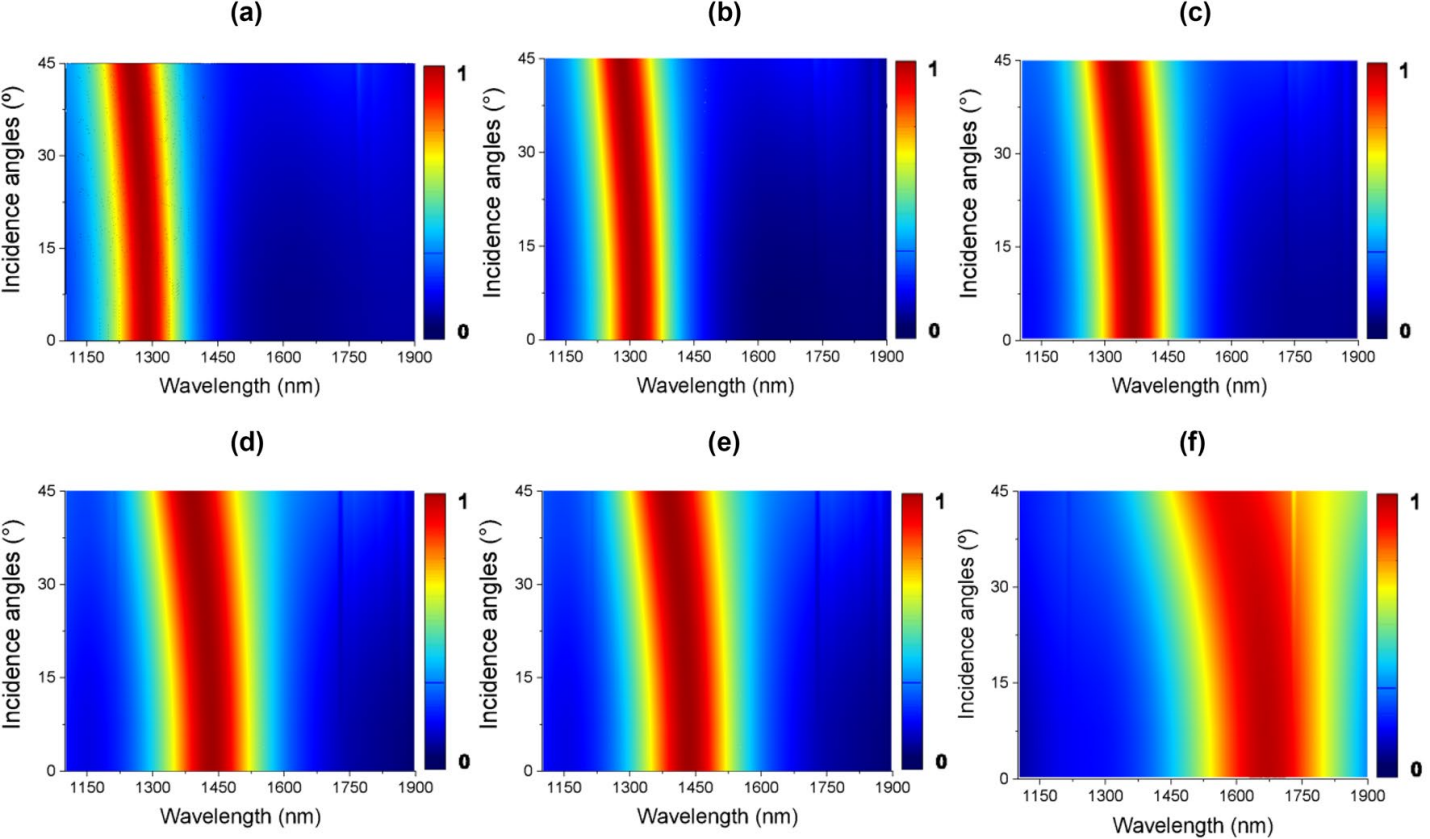
Dependence of the absorption for incident angles from 0° to 45° in TE polarization in the 1100–1900 nm spectrum on: (**a**) amorphous phase (*m* = 0%), (**b**) *m* = 20%, (**c**) *m* = 40%, (**d**) *m* = 60%, (**e**) *m* = 80%, and (**f**) crystalline phase (*m* = 100%).

The absorption responses as a function of the incident angle and crystallization phases of the PCM have been also analyzed. For TE polarization, the resonant wavelength remains constant up to 30°, then it shifts slightly to shorter wavelengths, as shown in Fig. 5a–f. The numerical results show absorption close to the unit for all analyzed phases for normal incidence (θ = 0°) and oblique angles of incidence up to 45°.

**Figure 6:**
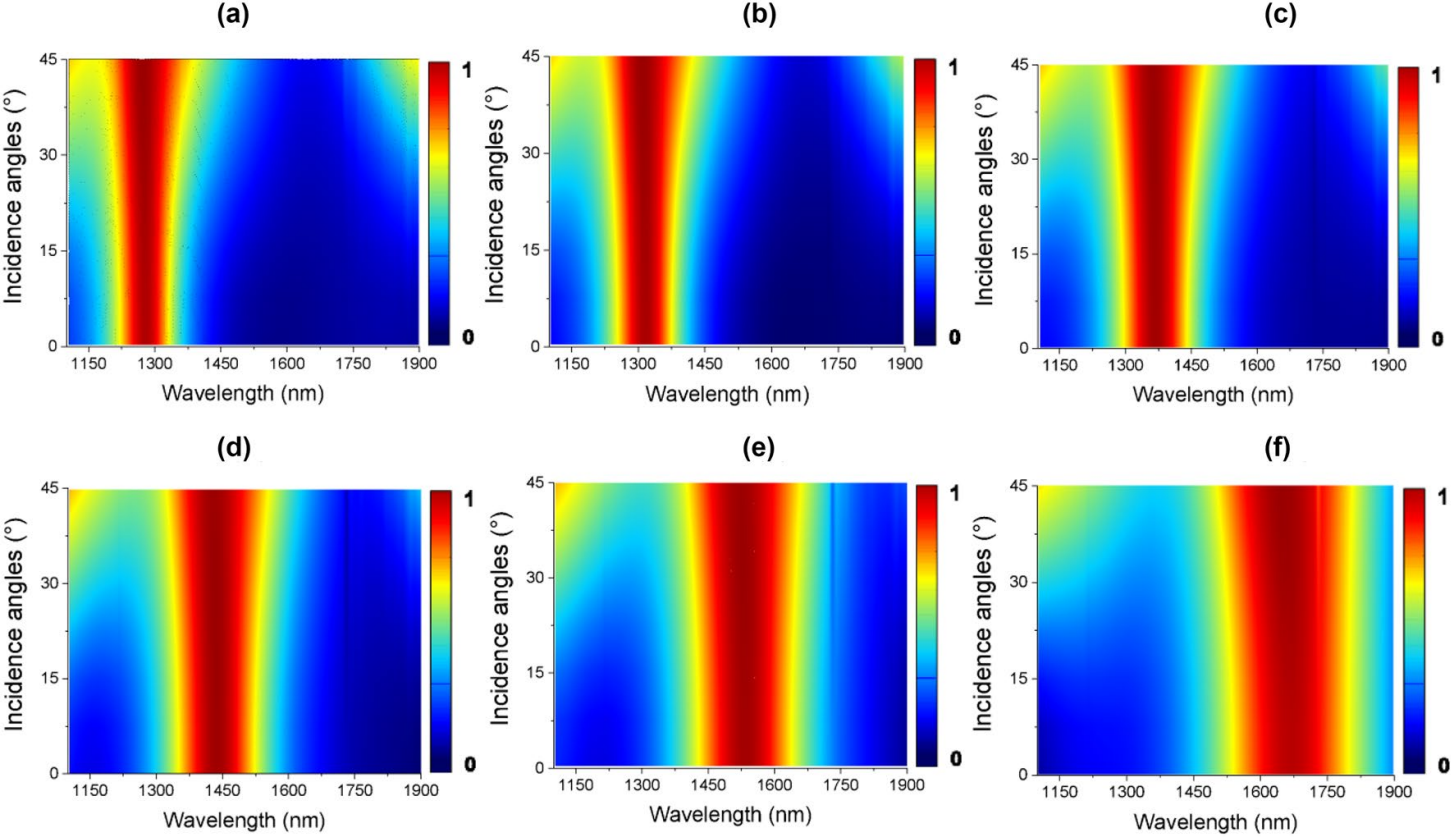
Dependence of the absorption for incident angles from 0° to 45° in TM polarization in the 1100–1900 nm spectrum on: (**a**) amorphous phase (*m* = 0%), (**b**) *m* = 20%, (**c**) *m* = 40%, (**d**) *m* = 60%, (**e**) *m* = 80%, and (**f**) crystalline phase (*m* = 100%).

The absorption performances for TM polarization are shown in Fig. 6a–f. The numerical results show high absorption for all presented angles (from 0° to 45°). It is possible to observe a great angular insensitivity, which is ideal for several practical applications^46–49^. The results also show that there is a high absorption at wavelengths close to the resonant peak, which increases as the incident angle increases. Thus, the proposed hybrid absorber behaves as wideangle insensitive polarization device across the infrared spectral region. The wideangle behavior of the proposed device can be explained by the subwavelength size of the nanoantenna which can act as a broadband impedance-matched structure^50^. The unitary cell of the proposed absorber is about 300 nm × 300 nm × 400 nm which is critical to achieve wideangle absorption. Numerical simulations^51^ have found that the A(θ) rapidly drops to small values when the unitary cell is large, in this way the incident angle of the electromagnetic radiation has low influence on the response of the absorber. Moreover, because only a unitary cell has been considered in the vertical direction with subwavelength thickness, a wideangle operation could be expected since narrow band absorbance will occur when several layers are stacked.

**Figure 7:**
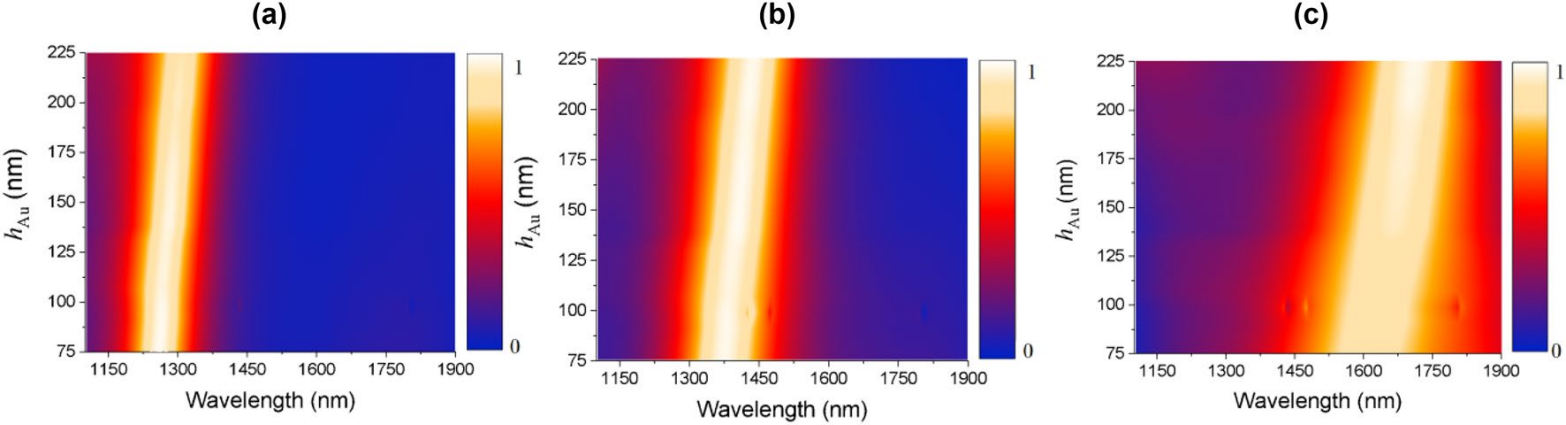
Absorption map in relation to the thickness of golden square (*h*_Au_) scanning for (**a**) amorphous; (**b**) semi-crystalline and; (**c**) crystalline phases.

To analyze the manufacturing tolerance of the device, we studied the absorption effects of the thickness of the golden squares (*h*_Au_). A sweep has been performed for widths between 75 and 225 nm, which are smaller and larger than the ideal values (150 nm). The results can be seen in Fig. 7a–c. and they demonstrate that the absorption remains high and the peak displacement is proportional to the variation in the thickness of the *h*_Au_ metallic layer. Furthermore, the peak shifting and the increasing of the FHWM caused by crystallization variation do not interfere with the absorption efficiency, even for considerably large or small thicknesses.

**Figure 8:**
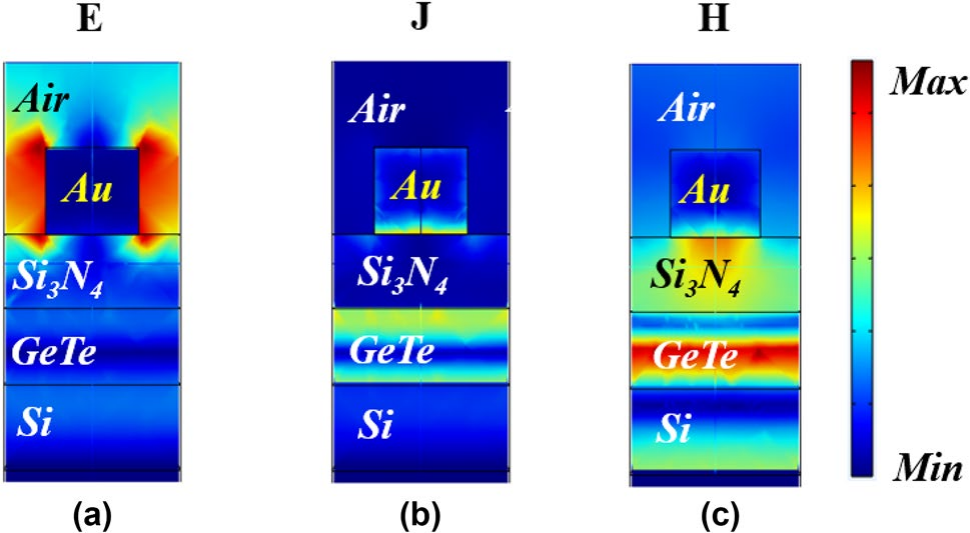
Spatial distribution of (**a**) normalized electric field; (**b**) normalized current density and; (**c**) normalized magnetic field of the absorber on resonant wavelength at 1550 nm and *m* = 82.5%.

In order to explain the physical mechanism of the absorber, we present the spatial distribution of the fields **E**, **H**, and the current density **J** in the xz plane at the resonant wavelength of 1550 nm, with a crystallization fraction of m = 82.5% (Fig. 8a–c). The results show an electric field strength between the side spacing of the top metallic layer. Current density is distributed between the semiconductor and the bottom side of the metallic layer. The intense magnetic field present in the PCM layer favors the interaction between the silicon nitride dielectric layer and the metallic layer, causing interference and generating SPP's, due to the semiconductor characteristic so that the absorption remains high. This constructive interference was caused by the choice of the physical and geometrical parameters of the structure for phase compensation, resulting in total absorption at the resonant peak. Spatial distribution of the absorption reveals that performance is higher at the interface between metal–dielectric^52^.

**Figure 9:**
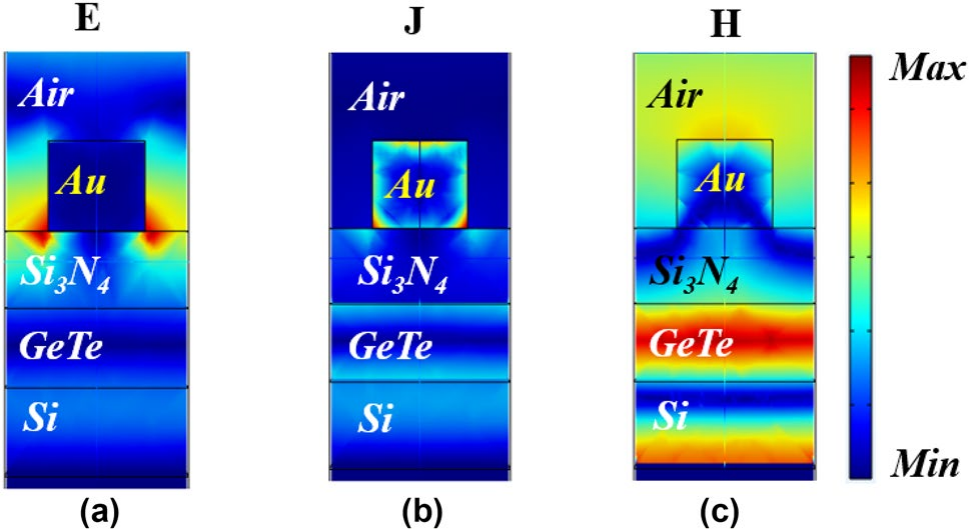
Spatial distribution of (**a**) normalized electric field; (**b**) normalized current density and; (**c**) normalized magnetic field of the absorber on resonant wavelength at 1550 nm and *m* = 0%.

Likewise, the spatial distributions of the fields of the normalized **E**, **J** and **H**, respectively, for the same wavelength (λ = 1550 nm), but with the PCM phase in an amorphous state and the results can be analyzed in Fig. 9a–c, therefore, non-resonant wavelength. The results reveal that there is no field weakening at the metal-air interface. Because of the GeTe’s magnetic field increases, the resonance is reflected at this point and shifted to shorter wavelengths^53^, according to the fitting Eq. (4). Current density is almost completely eliminated, concentrating on specific points on the upper layer.

**Figure 10:**
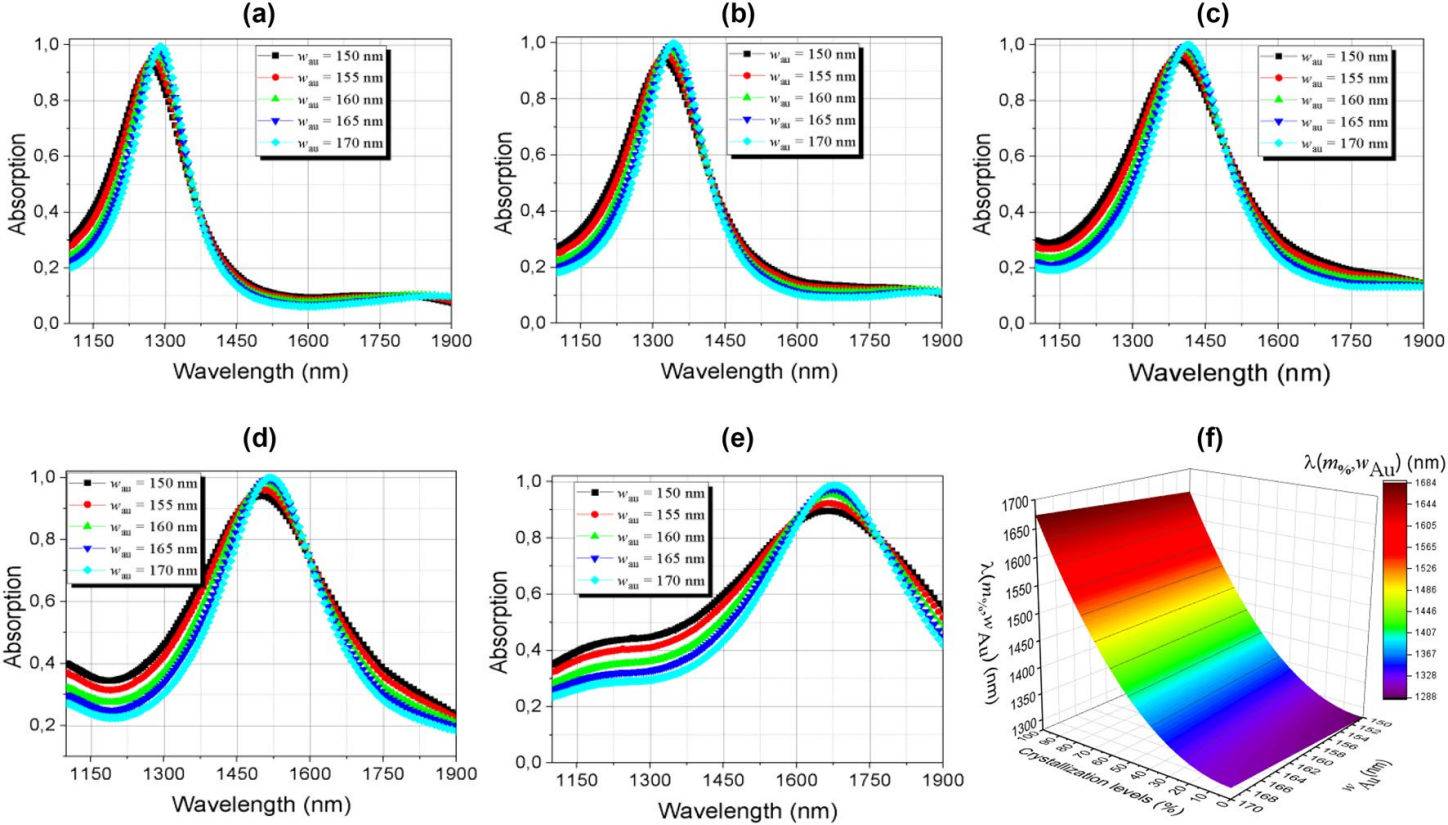
Absorption dependence of the *w*_Au_ metallic layer width in the range of 1100–1900 nm (**a**) amorphous; (**b**) semi-crystalline (m = 25%); (**c**) semi-crystalline (m = 50%); (**d**) semi-crystalline (m = 75%); (**e**) crystalline phases and; (**f**) surface graph of the multivariable function.

The effects of the width of the metallic layer of this absorber (*w*_Au_) were also analyzed for values in the interval 150–170 nm. Its peak resonance capability can be adjusted by shifting GeTe phases according to Fig. 10a–e. The results showed that there is a displacement of the resonance peak for longer wavelengths due to the increase in the layer width in all phases of GeTe. The increasing in the width of the gold nanoantenna results in a large area, since the current density is concentrated in this region (Fig. 8b) the absorption peak value increases.

Additionally, and based on methods of multiple linear regression, the curves were fitted to determine a function in which the fraction of crystallization of GeTe and the thickness of the metallic layer are independent variables and can be adjusted by the equation:

6 $$\uplambda (m_{\% } ,w_{Au} ) = 374.10^{ - 2} \;m^{2} + 976.10^{ - 2} \;w_{Au} + 1142,98$$

here, *m*_%_ is the crystallization fraction of GeTe and wAu the thickness of gold. For the analysis of the behavior of resonance peaks during evolution (phase change), a surface graph is presented in Fig. 10f, where these two independent variables have been combined.

**Figure 11:**
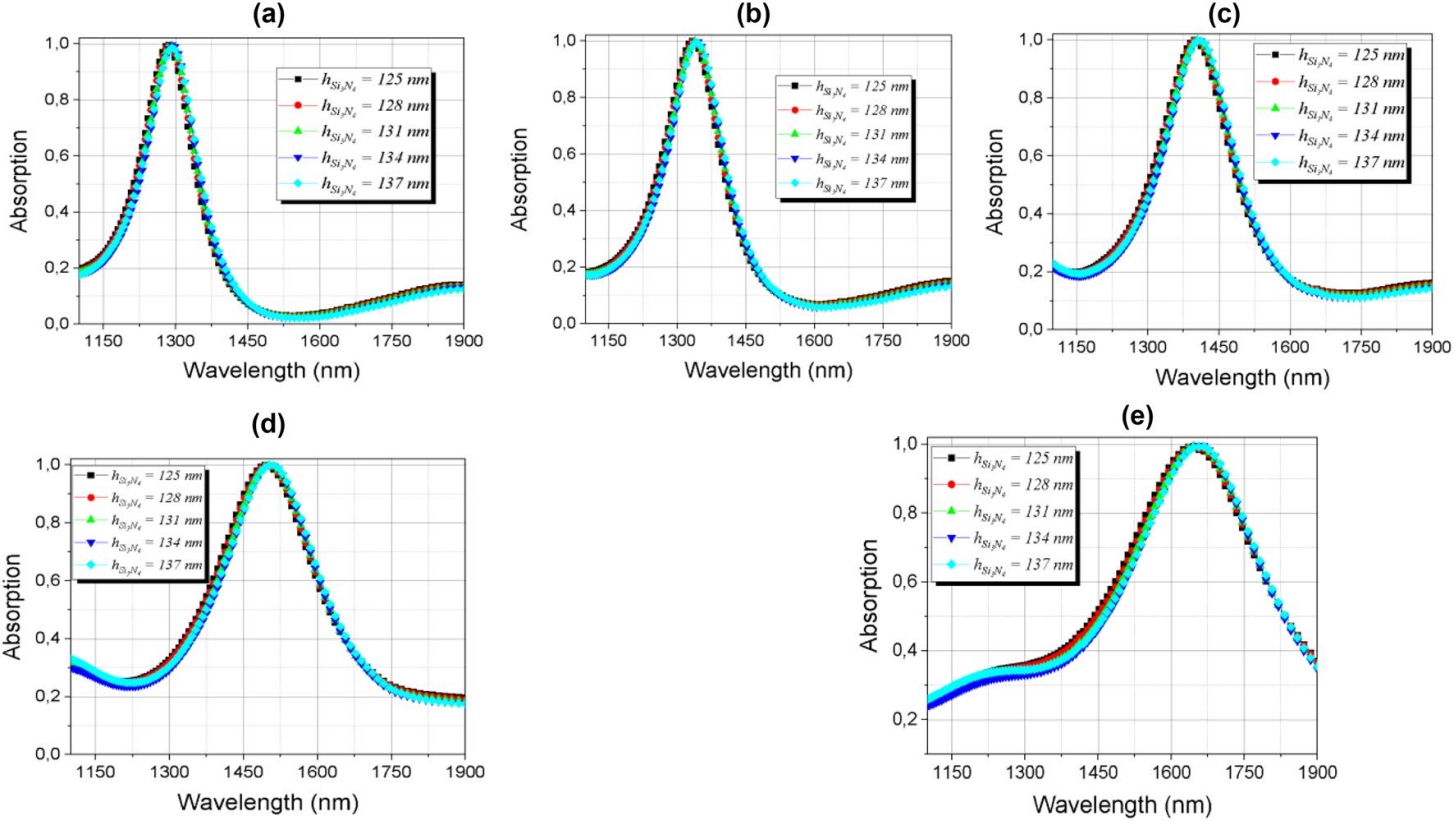
Absorption dependence of the dielectric thickness of Si_3_N_4_ *h*_Si3N4_ in the range of 1100–1900 nm (**a**) amorphous; (**b**) semi-crystalline (m = 25%); (**c**) semi-crystalline (m = 50%); (**d**) semi-crystalline (m = 75%); (**e**) crystalline phases.

The absorption effects have been also analyzed by varying the thickness of the *h*_Si3N4_ dielectric layer and their results are shown in Fig. 11a–e. The results show insensitivity to the variation of this geometric parameter. The variation of the thickness of the Si_3_N_4_ almost does not interfere with the absorption response because the highest current density is located in the gold nanoantenna base and in the PCM (GeTe) layer. For this reason there was no need for linearization. Despite this, absorption remained high for all phases of PCM.

**Figure 12:**
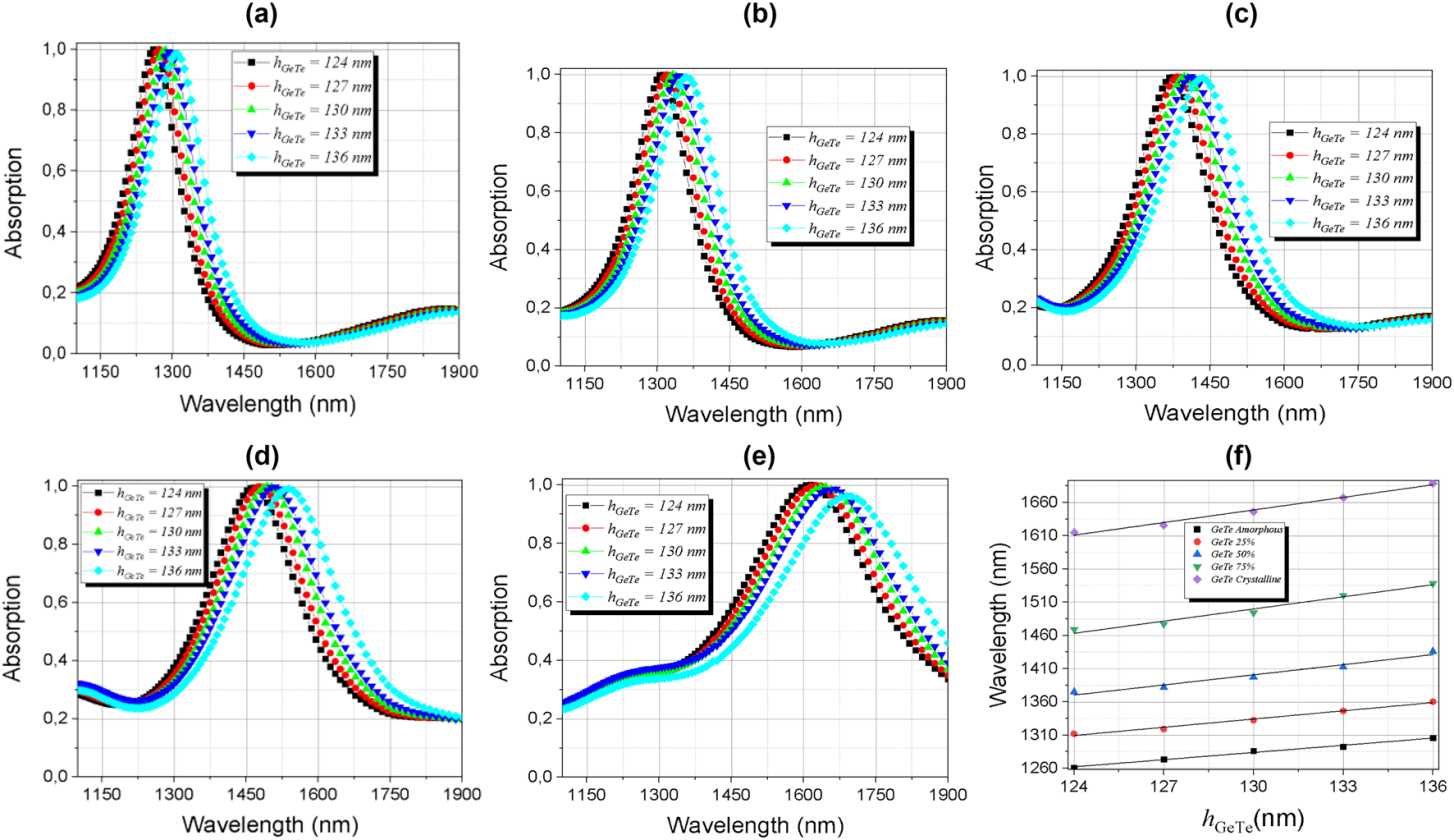
Absorption dependence of the PCM thickness *h*_GeTe_ in the range of 1100–1900 nm (**a**) amorphous; (**b**) semi-crystalline (m = 25%); (**c**) semi-crystalline (m = 50%); (**d**) semi-crystalline (m = 75%); (**e**) crystalline phases and (**f**) linear dependence of the peaks of (**a**–**e**) with the thickness of the GeTe layer (*h*_GeTe_).

The effects of absorption by varying the layer thickness of the material with phase change have been also investigated and the results are presented in Fig. 12a–e. The results show that increasing the PCM layer results in a shift of the resonant peak to higher wavelengths, due to the change in material thickness and optical modulation caused by crystallization. The results also reveal that these displacements are slightly spaced. The curves were linearized as can be seen in Fig. 12-f, making it possible to reach linear functions to adjust resonant wavelengths with the thickness of the PCM and the linear expressions are presented in Table 1.

**Table 1:** Linearization of the absorber resonant peaks with *h*_GeTe_.

GeTe’s phase change	Linear expression	
Amorphous (m = 0%)	$\uplambda (h_{{{\text{GeTe}}}} ) = 3.61\;h_{{{\text{GeTe}}}} + 814.79$	(7)
SemiCrystalline (m = 25%)	$\uplambda (h_{{{\text{GeTe}}}} ) = 4.14\;h_{{{\text{GeTe}}}} + 796.30$	(8)
SemiCrystalline (m = 50%)	$\uplambda (h_{{{\text{GeTe}}}} ) = 5.08\;h_{{{\text{GeTe}}}} + 740.45$	(9)
SemiCrystalline (m = 75%)	$\uplambda (h_{{{\text{GeTe}}}} ) = 6.11\;h_{{{\text{GeTe}}}} + 705.91$	(10)
Crystalline (m = 100%)	$\uplambda (h_{{{\text{GeTe}}}} ) = 6.32\;h_{{{\text{GeTe}}}} + 827.05$	(11)

**Figure 13:**
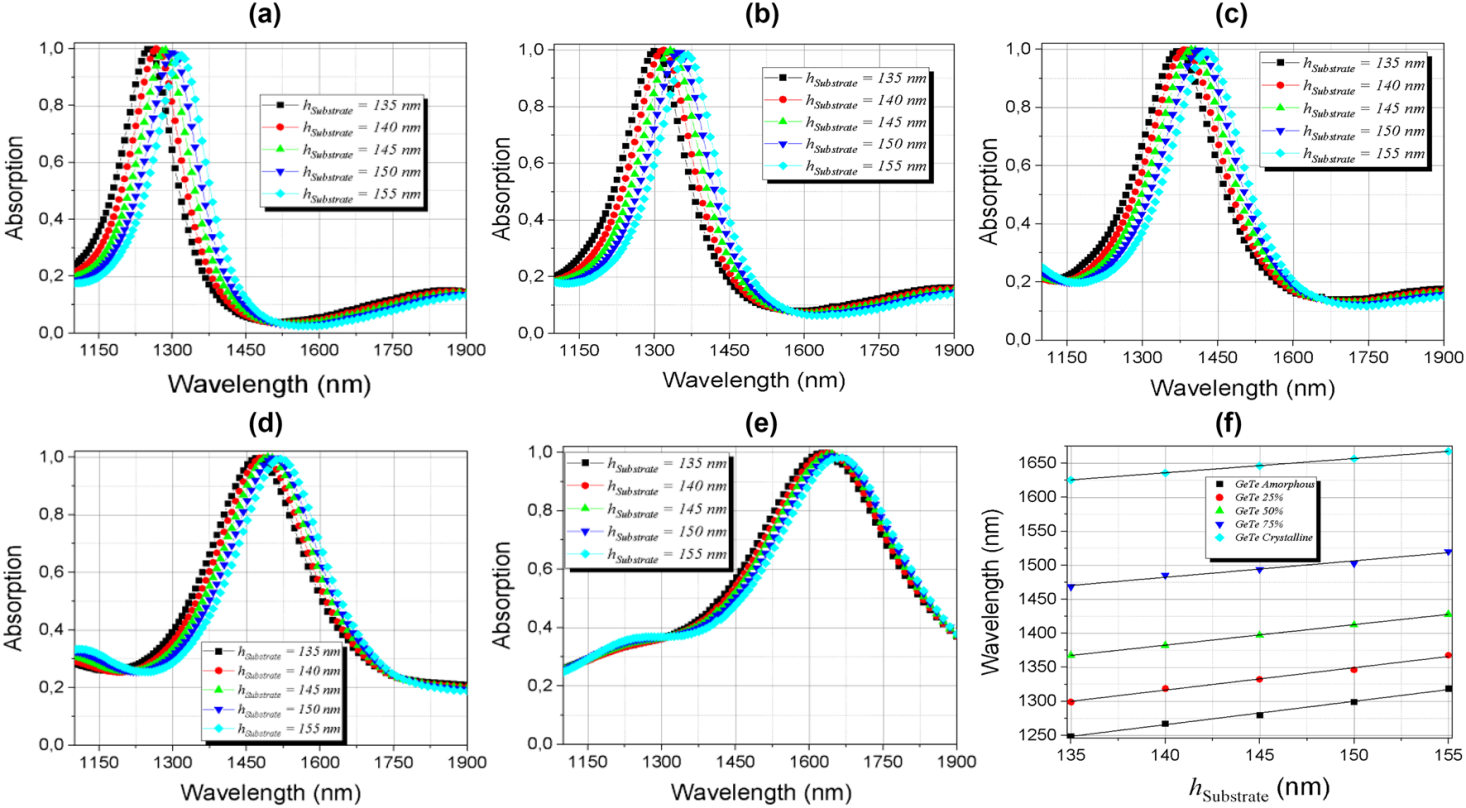
Absorption dependence of the silicon substrate thickness *h*_Substrate_ in the range of 1100 to 1900 nm (**a**) amorphous; (**b**) semi-crystalline (m = 25%); (**c**) semi-crystalline (m = 50%); (**d**) semi-crystalline (m = 75%); (**e**) crystalline phases and (**f**) linear dependence of the peaks of (**a**–**e**) with the thickness of the substrate thickness (*h*_Substrate_).

The effects on the absorption by varying the layer thickness of the silicon dielectric substrate material were also analyzed and the results are shown in Fig. 13a–e. They reveal that its variation interferes with absorption and that there is also a displacement of the resonant peaks. The curves were also linearly fitted, allowing the control of the peaks through linear functions shown in Table 2. The results of the linear dependence of the resonant peaks with the variation of the substrate layer thickness can be seen in Fig. 13-f. The thickness of the GeTe and silicon layers are related with the resonant cavity size, then, the resonance wavelength shift linearly to higher wavelength values when they increases.

**Table 2:** Linearization of the absorber resonant peaks with *h*_Substrate_.

GeTe’s phase change	Linear expression	
Amorphous (m = 0%)	$\uplambda (h_{Substrate} ) = 3.30\;h_{Substrate} + 854.78$	(12)
SemiCrystalline (m = 25%)	$\uplambda (h_{Substrate} ) = 3.30\;h_{Substrate} + 854.78$	(13)
SemiCrystalline (m = 50%)	$\uplambda (h_{Substrate} ) = 3.02\;h_{Substrate} + 959.54$	(14)
SemiCrystalline (m = 75%)	$\uplambda (h_{Substrate} ) = 2.42\;h_{Substrate} + 1143.70$	(15)
Crystalline (m = 100%)	$\uplambda (h_{Substrate} ) = 2.10\;h_{Substrate} + 1342.35$	(16)

**Figure 14:**
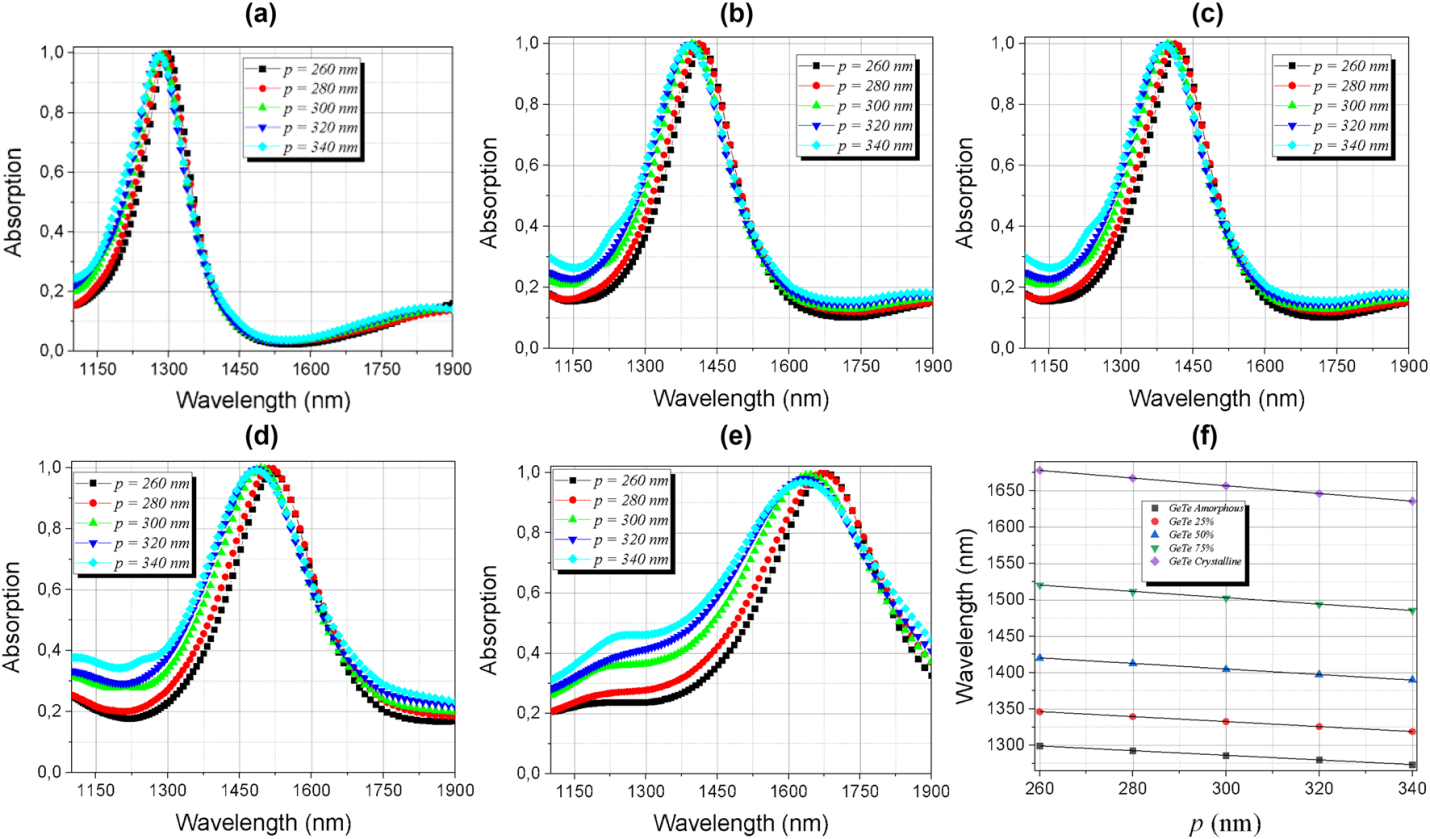
Absorption dependence of the periodicity *p* in the range of 1100 to 1900 nm (**a**) amorphous; (**b**) semi-crystalline (m = 25%); (**c**) semi-crystalline (m = 50%); (**d**) semi-crystalline (m = 75%); (**e**) crystalline phases and (**f**) linear dependence of the peaks of (**a**–**e**) with the thickness of the periodicity.

The dependence of the absorption as a function of the periodicity of the structure is also analyzed as can be seen in Fig. 14a–e. The results showed that the increase in periodicity results in a shifting of the resonant peak to shorter wavelengths, indicating a linear decrease, shown in Fig. 14-f. Based on the presented results, the curves were fitted, making it possible to control the resonance peaks in a function of the variation in the periodicity of the absorber, which can be analyzed in Table 3.

**Table 3:** Linearization of the absorber resonant peaks with the periodicity *p.*

GeTe’s phase change	Linear expression	
Amorphous (m = 0%)	$\uplambda (p) = - 0.32p + 1381.93$	(17)
SemiCrystalline (m = 25%)	$\uplambda (p) = - 0.34p + 1435.35$	(18)
SemiCrystalline (m = 50%)	$\uplambda (p) = - 0.38p + 1519.21$	(19)
SemiCrystalline (m = 75%)	$\uplambda (p) = - 0.43p + 1633.67$	(20)
Crystalline (m = 100%)	$\uplambda (p) = - 0.53p + 1815.92$	(21)

## Conclusion

In conclusion, we demonstrated a hybrid and multilayer three-dimensional resonant absorber, composed of chalcogenide phase change material (GeTe). The absorbers have been numerically analyzed and high optical modulation caused by the variation of the GeTe crystallization phase has been demonstrated. We numerically determined that the resonant peak wavelength can be controlled as a function of the crystallization fraction, as well as the physical parameters such as the thickness of the layers, periodicity and width of the upper gold layer. The resonant wavelength can be empirically obtained through linear, 3rd degree polynomials and multivariables expressions. The dependence of the structure on the incident angle up to 45° was also analyzed and the absorption remained high regardless of polarization. The excellent results remain constant even for variations in the thickness of the gold layer to account for possible errors in the manufacturing process. The physics mechanism of the absorption and the phase change of the chalcogenide allows it to be used as a semiconductor and insulator, and this reconfigurability modify the concentration of the electromagnetic fields, displacing the structure's resonance peaks to others regions. We would consider the use of approaches based on neuromorphic systems in future proposals^54^. This structure can be an alternative solution for absorbers with potential applications for various technologies employed in reconfigurable nanophotonic devices.
